# Supplementary material for: Practitioners’ Views on Nicotine Replacement Therapy in Pregnancy during Lapse and for Harm Reduction: A Qualitative Study
Source: Int J Environ Res Public Health. 2019 Nov 29;16(23):4791. doi: 10.3390/ijerph16234791 (PMC6926779; doi:10.3390/ijerph16234791)
Supplement: Supplementary file 1 [file ijerph-16-04791-s001.zip › Supplementary Materials, S1 FG1 topic guide.docx]

Focus Group 1 Topic Guide

**Q1. How do you all maximise pregnant women’s take up and subsequent engagement with the use of NRT at the first contact they have with smoking cessation services?**

**Prompts**

- Tips
- What works what doesn’t work
- Motivation
- Pressure to say yes (from interviews)
- Frequent concerns they tell you about
- Women’s expectations

**Q1A. How do you encouraging women to start using NRT without putting them off?**

**Prompts**

- Is it a first contact issue?

**Q.2.How do you think the giving out of all relevant information on NRT should be managed?**

**Prompts**

- Overload
- when
- Ideal times -drip feed

- 1 go

-Relevant points

- Long term management
- Most effective approach? Why?
- How do you answer any questions/concerns?
- Pick up on deviations from advice

**Q2A. What methods of delivery of information on NRT are best at these times?**

**Prompts**

- Face-to-face
- Leaflets
- Texts
- Phone call
- Web site
- apps
- other
- strengths and weaknesses of the different methods?
- Ideas on improving

**Q.3. How do you go about countering misinformation about NRT in your regular practice?**

**Q.4: how do you tackle using NRT during pregnant women’s brief lapse or full relapse to smoking to help them becoming abstinent from smoking again or to stop smoking permanently?**

**Prompts**

- Advice you give
- Best for giving up completely
- Differences between lapse and relapse?

**Q.5 Drawing on your experiences what would you include in a Rolls Royce support package of care for pregnant women on their journey with NRT in-between or after consultations with practitioners?**

**Prompts**

- Key messages to include
- timing
- format
- Practical vs emotional
- content
- Do/should women have choices on delivery
- why

**Q.6 Please discuss/share with each other, how you are dealing with this issue of vaping and NRT combined with your clients**

**Q.7 Are any of you seeing/working on, opportunities in these changes, for improving women’s experiences of using NRT?**

**Prompts**

- Technology
- If not, why not

**Q.8: We have presented you with up to date research on evidence for efficacy & safety of NRT**. What are your views on NRT use and dosing with regards to:

1. Offering dual NRT
2. Offering NRT for whole of pregnancy
3. Continuing NRT during brief lapses or ‘full blown’ relapse
4. Offering NRT even if woman does not want to stop smoking
