# Supplementary material for: Practitioners’ Views on Nicotine Replacement Therapy in Pregnancy during Lapse and for Harm Reduction: A Qualitative Study
Source: Int J Environ Res Public Health. 2019 Nov 29;16(23):4791. doi: 10.3390/ijerph16234791 (PMC6926779; doi:10.3390/ijerph16234791)
Supplement: Supplementary file 1 [file ijerph-16-04791-s001.zip › Supplementary Materials, S2 FG2 topic guide.docx]

**Focus Group 2 topic guide**

**Issue 1**: Addressing misinformation

**Essence of message:** You can trust what we are saying

**Example Message:** Regardless of what you may hear from other people or read about using NRT while you are pregnant what we are recommending is based on the latest scientific findings and advice.

**Prompts:**

- How would you try to persuade women to trust and believe that information they are given is correct
- how confident would you be in delivering this message

What methods do you think would be useful in helping deliver this message?

**Issue 2**: Stopping NRT too early

**Essence of message:** Don’t be over confident and don’t stop NRT until you have spoken to an advisor

**Example Message:** You can’t easily tell when NRT is working. If NRT is doing its job then you probably won’t notice it but don’t let this trick you into thinking you’ve quit already and you don’t need to keep taking it. It’s important to take the NRT for as long as recommended and don’t stop your NRT until you have spoken to your stop smoking practitioner

**Prompts:**

- Research suggests this is potentially an important message …..what do you think?
- How do you know if it is time for women to stop taking NRT
- Is it be appropriate for some individuals to continue for longer? How might you assess this?
- What methods do you think would be useful in helping deliver this message

**Issue 3**: Addressing women’s concerns about NRT safety

**Essence of message:** NRT won’t harm your baby

**Example Message:** There is no evidence that NRT causes harm to your baby

Or

NRT has never been shown to cause harm to babies

**Prompts:**

- Research suggests this is potentially an important message …..what do you think?
- Which message do you prefer?
- Would you feel confident in delivering this message?

What methods do you think would be useful in helping deliver this message

**Issue 4**: Addressing women’s concerns about NRT safety

**Essence of message:** It is OK to use NRT throughout your pregnancy

**Example Message:** It is OK to use NRT throughout your pregnancy

**Prompts:**

- Research suggests this is potentially an important message …..what do you think?
- Would you feel confident in delivering this message?
- How would you try to persuade women / deliver this message in your consultations?
- What methods do you think would be useful in helping deliver this message

**Issue 5**: Addressing women’s concerns about getting too much nicotine

**Essence of message:** NRT gives you less nicotine than smoking

**Example Message:** NRT gives you a lot less nicotine than you would have received by smoking

**Prompts:**

- Research suggests this is potentially an important message …..what do you think?
- Would you feel confident in delivering this message?
- How would you try to persuade women / deliver this message in your consultations?
- What methods do you think would be useful in helping deliver this message

**Issue 6**: Addressing women’s concerns about getting too much nicotine

**Essence of message:** Even if you use NRT and smoke you will probably be exposed to no more nicotine than smoking alone

**Example Message:** Research shows, If you try stopping smoking with NRT but lapse and smoke a little, the baby will be exposed to less tobacco smoke and probably no more nicotine than just smoking

**Prompts:**

- what do you think of this message? Is it useful?
- Would you feel confident in delivering this message?
- How would you try to persuade women / deliver this message in your consultations?
- What methods do you think would be useful in helping deliver this message

**Issue 7**: Addressing women’s concerns about becoming addicted to NRT

**Essence of message:** You are not likely to get addicted to NRT

**Example Message:** Using NRT is not trading one nicotine addiction for another. The way NRT is delivered makes it much less addictive than smoking and long-term dependence on NRT, even in high doses, is highly unlikely

**Prompts:**

- what do you think of this message?
- Do you deliver this message in your practice
- Would you feel confident in delivering this message?
- How would you try to persuade women / deliver this message in your consultations?
- What methods do you think would be useful in helping deliver this message

**Issue 8**: Stopping NRT during a lapse

**Essence of message:** Don’t stop the NRT even if you do smoke a little

**Example Message:** If you do start smoking for a short time do not stop using the NRT (even if you continue to smoke a little). Re-commit to stopping and you can get back on track and stop smoking

**Prompts:**

- what do you think of this message? How would you define lapse/short time?
- Do you deliver this message in your practice
- Would you feel confident in delivering this message?
- How would you try to persuade women / deliver this message in your consultations?
- What methods do you think would be useful in helping deliver this message

**Issue 9**: Information provision

**Essence of message:** the rate of nicotine metabolism is increased in pregnancy

**Example Message:** Nicotine is removed from your body a lot more quickly when you are pregnant; this means you need higher doses of nicotine to prevent cravings.

**Prompts:**

- Research suggests is may be an important message of which interviewed women tell us they are unaware. Do you think it’s important or not?
- Would you feel confident in delivering this message?
- How would you try and get this message across to women?
- What methods do you think would be useful in helping deliver this message

**Issue 10**: Some women are unsure why they are getting dual therapy and worried about getting too much nicotine

**Essence of message:** It’s safe to use two products, you won’t be getting too much nicotine

**Example Message:** Because nicotine is removed from your body a lot more quickly when you are pregnant you need two NRT products to prevent cravings. This is safe for the baby.

**Prompts:**

- Research suggests This is may be an important message of which interviewed women tell us they are unaware. Do you think it’s important or not?
- Would you feel confident in delivering this message?
- How would you try and get this message across to women?
- What methods do you think would be useful in helping deliver this message

**Issue 11**: Sometimes women confuse nicotine withdrawal–when they need more nicotine–with side effects from too much nicotine and stop NRT when they should be taking more.

**Essence of message:** These withdrawal symptoms are a sign you need more NRT not a reason to stop

**Example Message:** You may find that you experience some of the following nicotine withdrawal symptoms; restlessness, irritability, frustration, tiredness, hunger, difficulty sleeping or concentrating. These are a sign you may need more NRT not a reason to stop your NRT

**Prompts:**

- Is this something you’ve noticed (i.e. women confusing W/D with nicotine side effects / overload)?What WD are reported the most?

- Is this an important issue to address or not?
- Would you feel confident in delivering this message?
- How would you try and get this message across to women?

What methods do you think would be useful in helping deliver this message

**Issue 12**: Help women differentiate between withdrawal and side effects of nicotine

**Essence of message:** These are the side effects of nicotine

**Example Message:** The side effects of NRT are dizziness; headache; excessive sweating; nausea; palpitations; skin reactions; vomiting. You may experience these if you use too much NRT but this is very unlikely.

**Prompts:**

- Is this something you’ve noticed (i.e. women confusing W/D with nicotine side effects / overload)? What SE are reported the most?

- Is this an important issue to address or not?
- Would you feel confident in delivering this message?
- How would you try and get this message across to women?

What methods do you think would be useful in helping deliver this message

**Issue 13**: Women are concerned about NRT side effects

**Essence of message:** That NRT product side effects are nothing to worry about

**Example Message:** Like medicines, NRT products can have side-effects they are typically mild, don’t get worse, and usually disappear. They are not a sign of anything bad

**Prompts:**

- Is this an important issue to address or not?
- Do you deliver similar?
- Would you feel confident in delivering this message?
- How would you try and get this message across to women?
- How do you help women deal with these side effects

What methods do you think would be useful in helping deliver this message
